# International multidisciplinary expert consensus on the clinical application of patient-specific subperiosteal implants: a modified Delphi study

**DOI:** 10.1186/s40729-026-00729-8

**Published:** 2026-10-26

**Authors:** Yong-Dae Kwon, Anne-Kathrin Bär, Momen A. Atieh, Bilal Al-Nawas

**Affiliations:** 1 Department of Oral and Maxillofacial Surgery, College of Dentistry, Kyung Hee University, Seoul, Republic of Korea; 2 Department of Oral and Maxillofacial Surgery, University Medical Centre Mainz, Augustusplatz 2, 55131 Mainz, Germany; 3 Department of Oral and Maxillofacial Surgery, Federal Armed Forces Hospital, Koblenz, Germany; 4 Mohammed Bin Rashid University of Medicine and Health Sciences, Hamdan Bin Mohammed College of Dental Medicine, Dubai Healthcare City, Dubai, United Arab Emirates; 5 Sir John Walsh Research Institute, Faculty of Dentistry, University of Otago, Dunedin, New Zealand; 6 School of Dentistry, University of Jordan, Amman, Jordan

**Keywords:** Patient-specific subperiosteal implants, Delphi method, Severe jaw atrophy, Implant-supported prostheses, Computer-aided design, Consensus

## Abstract

**Purpose:**

To establish international multidisciplinary expert consensus on the clinical application of patient-specific subperiosteal implants across indications, surgical workflow, prosthetic rehabilitation, outcomes and complications, and follow-up and maintenance.

**Methods:**

A modified two-round web-based Delphi study was conducted in accordance with established methodological guidance and ACCORD. Evidence-informed preliminary statements were developed by a multidisciplinary core group. International experts rated each statement on a five-point Likert scale and could provide comments. Consensus was defined as ≥ 75% agreement. Non-consensus or substantively revised statements were redistributed in Round 2.

**Results:**

Of 58 invited experts, 23 participated in Round 1 (39.7% response); 22 returned for Round 2 (95.7% retention), with 21 providing valid statement-level ratings. In Round 1, 30 of 43 statements (69.8%) achieved consensus. Round 2 evaluated 15 revised statements (13 non-consensus and two substantively refined), of which 12 (80.0%) achieved consensus. The final set comprised 40 consensus statements across five domains. Agreement was strongest for surgical workflow and follow-up and maintenance, whereas uncertainty persisted regarding indications and outcomes and complications. No consensus was reached on previous head and neck radiotherapy, locking screw fixation systems, or the frequency of mechanical complications.

**Conclusions:**

This study provides an evidence-informed multidisciplinary framework for the clinical use of patient-specific subperiosteal implants. It supports careful patient selection, digitally integrated and prosthetically driven planning, individualized rehabilitation, and structured long-term follow-up, while identifying priorities for further clinical research.

**Graphical Abstract:**

**Figure:**
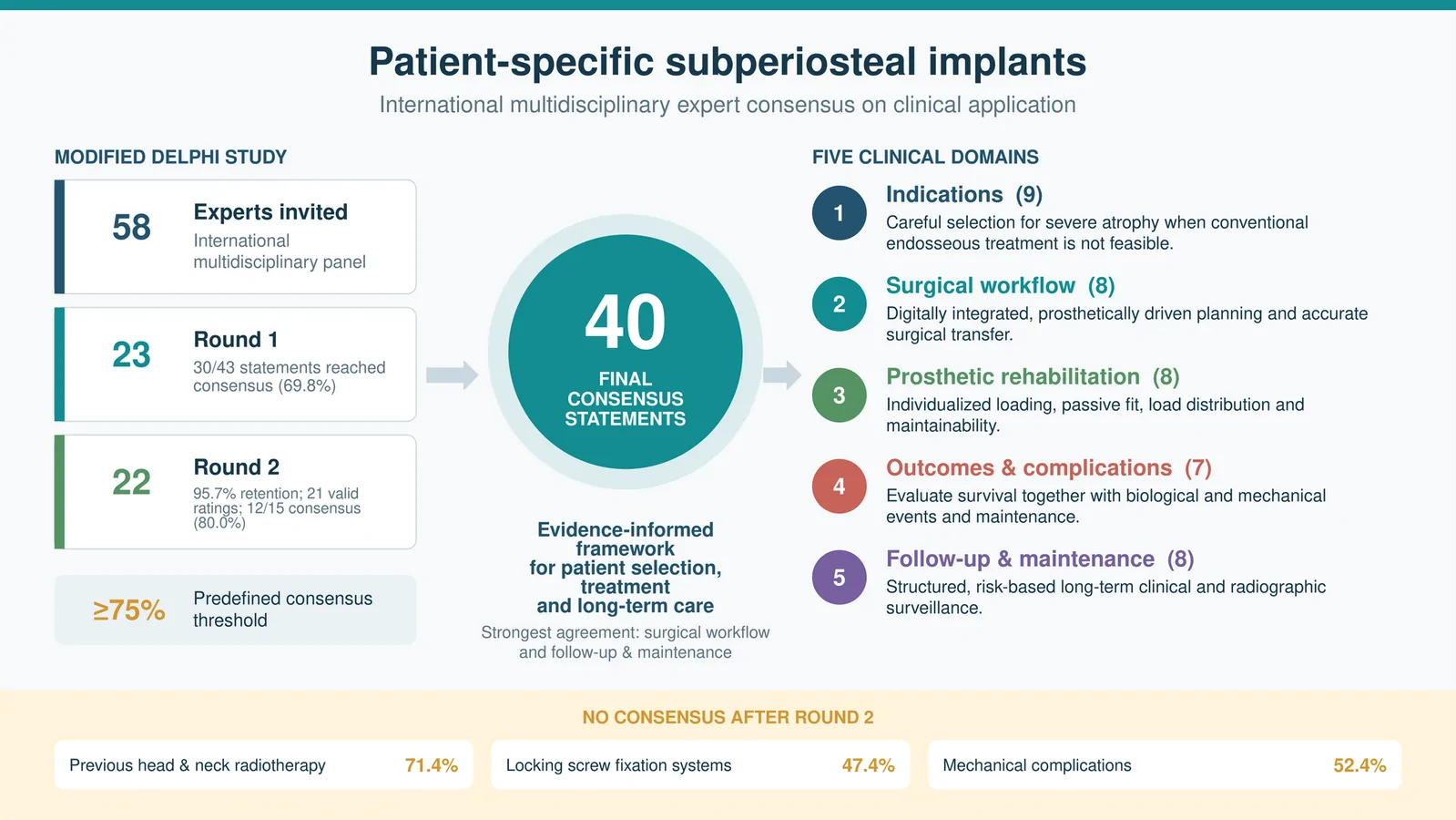

**Supplementary Information:**

The online version contains supplementary material available at https://doi.org/10.1186/s40729-026-00729-8.

## Introduction

Severe maxillary and mandibular atrophy remains a major challenge in implant rehabilitation when residual bone volume or anatomy precludes predictable placement of conventional endosseous implants. In these situations, treatment often requires extensive bone augmentation or alternative graftless approaches, which may increase surgical complexity, treatment duration, and morbidity in selected patients [1, 2]. The feasibility of these approaches may additionally be limited by the residual anatomy, previous treatment failure, medical comorbidity, or patients’ willingness to undergo staged reconstructive procedures. Patient-specific subperiosteal implants have therefore re-emerged as a potential treatment option for carefully selected patients in whom conventional implant therapy is not feasible or would require extensive reconstructive procedures.

Unlike historical subperiosteal implants, which were fabricated using direct bone impressions and were frequently associated with poor adaptation and biological complications, contemporary designs utilize cross-sectional imaging, virtual surgical planning, computer-aided design and manufacturing (CAD/CAM), and additive manufacturing. These technologies enable customized implant frameworks to be designed according to individual skeletal anatomy and prosthetic requirements while facilitating accurate fit, planned fixation, and prosthetically driven rehabilitation [3–5]. The evolution from these historical devices to contemporary patient-specific designs, together with the biological reasons for the high failure rates observed with earlier subperiosteal implants, has recently been summarized in detail [6]. Their reported applications have expanded beyond full-arch rehabilitation of severely atrophic jaws to include segmental defects, congenital craniofacial conditions, salvage procedures, post-oncological reconstruction and other complex clinical situations [4, 7–11].

Clinical outcomes are encouraging but must be interpreted in relation to the maturity and heterogeneity of the available evidence. Al-Nawas and Bär [7] reported in a systematic review of 24 publications, a survival rate of 97.6% after a mean follow-up of approximately 17 months. A subsequent scoping review mapped 116 contemporary publications but identified only 56 human clinical records; the evidence remained dominated by case reports, case series, and heterogenous observational cohorts, with few comparative studies and limited follow-up beyond five years [12]. Recent longer-term studies and evidence syntheses have further demonstrated that implant retention does not necessarily represent clinical success, particularly when soft-tissue exposure, inflammation, prosthetic complications, revision procedures, and maintenance requirements are considered [5, 13–15].

Despite the rapidly expanding clinical literature, standardized guidance for patient-specific subperiosteal implants remains limited. A previous consensus report and several recent reviews have addressed selected indications, technical principles, or outcomes, but, to our knowledge, no international multidisciplinary Delphi study has systematically evaluated the full clinical pathway from patient selection and surgical workflow to prosthetic rehabilitation, complication assessment, and long-term maintenance [12, 16]. Important uncertainties therefore remain regarding the relationship of patient-specific subperiosteal implants to established treatment alternatives, their use in medically compromised or previously irradiated patients, fixation and loading strategies, the interpretation of implant exposure and mechanical events, and appropriate follow-up protocols.

Against this background, where high-quality comparative evidence is limited, structured expert consensus provides a transparent method for integrating the best available evidence with multidisciplinary clinical expertise and helps to translate it into practical guidance. The modified Delphi method is particularly well suited to this purpose because it enables anonymous expert evaluation, structured feedback, and iterative refinement of recommendations while minimizing the influence of dominant individuals [17, 18].

Therefore, this modified Delphi study aimed to establish international multidisciplinary expert consensus on the clinical application of patient-specific subperiosteal implants across the domains of indications, surgical workflow, prosthetic rehabilitation, outcomes and complications, and follow-up and maintenance. The resulting consensus statements are intended to provide an evidence-informed framework to support clinical decision-making, promote greater consistency in practice, and identify priorities for future research.

## Materials and methods

A modified two-round Delphi study was conducted to establish international multidisciplinary expert consensus on the clinical application of patient-specific subperiosteal implants. The study was designed, conducted, and reported in accordance with the recommendations of Diamond and colleagues [17] and the Accurate Consensus Reporting Document (ACCORD) guideline for reporting consensus-based research [18]. The Delphi process was designed to conclude after two sequential web-based rounds.

### Development of Delphi statements

A multidisciplinary core development group developed the initial Delphi statements. To support statement development, a structured literature search was undertaken to identify contemporary evidence relating to patient-specific subperiosteal implants. The purpose of this search was to identify key clinical themes, areas of uncertainty, emerging technologies, and knowledge gaps relevant to the development of consensus statements, rather than to undertake a formal systematic review.

Electronic searches were performed in PubMed/MEDLINE, EMBASE, and the Cochrane Central Register of Controlled Trials (CENTRAL) via Ovid. Additional sources included ClinicalTrials.gov, ProQuest Dissertations and Theses Global, reference lists of relevant publications, and grey literature. Searches covered the period from 2000 to December 19, 2025. The search strategy incorporated terms relating to patient-specific implants, subperiosteal implants, three-dimensional subperiosteal implants, atrophic jaws, and digital planning. Search strategies were organized according to the five predefined thematic domains of the Delphi study: Indications, Surgical Workflow, Prosthetic Rehabilitation, Outcomes and Complications, and Follow-up and Maintenance. The complete database-specific search strategies are provided in Supplementary Table S1.

The evidence identified through the literature search, together with multidisciplinary discussions within the development group, guided the formulation of the preliminary statements. The draft statements underwent iterative review within the development group to improve clarity, eliminate ambiguity and redundancy, ensure comprehensive coverage of clinically relevant topics, and address areas of clinical uncertainty, innovation, and research gaps. Before dissemination, the questionnaire underwent internal pilot testing to assess statement clarity, relevance, content validity, feasibility, and suitability for expert evaluation.

### Participants and recruitment

An international multidisciplinary expert panel comprising practice- and research-based specialists in oral and maxillofacial surgery, periodontology, prosthodontics, biomaterials, biomedical engineering, and related disciplines was invited to participate. Purposive sampling was used to ensure that participants possessed relevant expertise in patient-specific subperiosteal implants, represented diverse geographical regions, and reflected multidisciplinary perspectives [19]. Eligible participants were required to have recognized experience in the field through clinical practice, research, education, device design and development, or other relevant professional activities.

Potential participants were identified by the core development group based on their documented professional expertise in the field and were invited individually by email. No formal sample size calculation was performed, in accordance with recommendations from the COMET initiative [20]. Instead, the focus was on attaining diverse representation from essential stakeholder groups while maintaining transparency in the consensus-building process [21]. Based on published methodological guidance and previous Delphi studies, the development group aimed to recruit approximately 25–35 experts, anticipating that around 30 participants would complete the first Delphi round and allowing for some attrition between rounds [22]. A between-round retention rate of at least 70% was considered acceptable to minimize the risk of attrition bias.

### Delphi questionnaire and survey administration

Both Delphi rounds were administered electronically using SurveyMonkey^®^ (Momentive Inc., San Mateo, CA, USA), with Round 1 conducted in May 2026 and Round 2 in July 2026. The Round 1 questionnaire comprised structured statements organized into five thematic domains. Participants rated each statement using a five-point Likert scale ranging from strongly disagree [1] to strongly agree [5]. An “unable to assess” response option was available and was excluded from the agreement denominator. Optional free-text fields allowed participants to explain their ratings, suggest modifications, identify ambiguities, and provide additional comments.

Participants received an invitation by email, and two reminder emails were sent before the closure of each survey round. Only participants who submitted a Round 1 response were invited to participate in Round 2.

Following Round 1, quantitative agreement and qualitative participant feedback were reviewed by the development group. Participant comments were used to identify ambiguities, refine wording, separate compound statements, consolidate overlapping concepts, and address issues raised during the first round. Statements that did not achieve consensus in Round 1, as well as statements that achieved consensus but required substantial revision or additional clarification based on participant feedback, were included in the Round 2 questionnaire.

Before Round 2, participants received a feedback report summarizing the aggregated Round 1 results, anonymized participant comments, and the rationale for any modifications made to the statements before re-rating them. Participants rated the revised statements using the same response scale and were again able to provide optional free-text comments.

### Consensus definition and statement disposition

Consensus was predefined as ≥75% of participants selecting “agree” or “strongly agree” (Likert scores of 4 or 5), consistent with published recommendations for Delphi methodology [17]. Agreement percentages were calculated using the number of participants providing a valid Likert-scale response for each statement as the denominator; responses marked as “unable to assess” or missing were excluded from the agreement denominator. Median Likert scores and interquartile ranges were reported descriptively, but did not determine whether consensus had been achieved.

Statements that achieved consensus in Round 1 and did not require substantial revision were accepted as final consensus statements and were not redistributed in Round 2. Statements that failed to achieve consensus or underwent substantial revision were included in the Round 2 questionnaire for re-evaluation. Following Round 2, statements achieving the predefined consensus threshold were accepted as final consensus statements, whereas those failing to achieve consensus were classified as non-consensus statements.

Minor editorial refinements were permitted after Round 2 solely to improve clarity or precision where explicitly supported by Round 2 participant comments and without altering the substantive meaning or scope of the statements; such refinements were not re-rated. Post-Round 2 editorial refinements and the participant comments supporting these changes are documented in Supplementary Table S2.

### Statistical analysis

Descriptive statistics were used to summarize participant demographics and Delphi responses. Median Likert scores, interquartile ranges (IQRs), and percentage agreement were calculated for each statement using IBM SPSS Statistics for Windows, Version 29.0 (IBM Corp., Armonk, NY, USA). SurveyMonkey summary reports were used to facilitate verification of agreement percentages and review of participant comments between Delphi rounds. Qualitative participant comments were used to support interpretation of quantitative findings and guide refinement of statements between Delphi rounds.

### Ethical considerations

Participation was voluntary, and electronic informed consent was obtained prior to accessing the questionnaire. The study collected professional opinions from expert participants only and did not involve patient recruitment or patient-level clinical data. Participant responses were anonymized during analysis and reporting.

## Results

### Participant characteristics and retention

Of the 58 experts invited, 23 completed the Round 1 questionnaire, corresponding to a response rate of 39.7%. All 23 participants provided at least one valid statement-level rating and were included in the Round 1 analysis. All Round 1 participants were invited to Round 2; 22 returned the questionnaire, of whom 21 provided at least one valid statement-level rating. Survey-level retention between rounds was therefore 95.7% (22/23).

Participant characteristics are summarized in Table 1. The panel included 15 oral and maxillofacial surgeons (65.2%), six implant dentists (26.1%), one periodontist (4.3%), and one prosthodontist (4.3%). Seventeen participants (73.9%) practiced in Europe, three (13.0%) in North America, one (4.3%) in Oceania, and one (4.3%) in the Middle East; geographical information was unavailable for one participant. Thirteen participants (56.5%) reported more than 20 years of professional experience, 12 (52.2%) had experience with more than 50 patient-specific subperiosteal implant cases, and 16 (69.6%) reported advanced experience with digital workflows.

**Table 1:** Characteristics of the round 1 Delphi panel (*N* = 23)

Characteristic	*n* (%)
**Age group, years**	
< 35	2 (8.7)
35–44	7 (30.4)
45–54	5 (21.7)
55–64	5 (21.7)
> 65	4 (17.4)
**Gender**	
Male	20 (87.0)
Female	3 (13.0)
Prefer not to say	0 (0.0)
**Primary discipline**	
Periodontics	1 (4.3)
Oral and maxillofacial surgery	15 (65.2)
Prosthodontics	1 (4.3)
Implant dentistry	6 (26.1)
Professional setting*	
Private practice	11 (47.8)
Academic institution	10 (43.5)
Hospital-based practice	2 (8.7)
Industry	0 (0.0)
Other	1 (4.3)
**Geographical region†**	
Europe	17 (73.9)
North America	3 (13.0)
Oceania	1 (4.3)
Middle East	1 (4.3)
Not reported	1 (4.3)
**Clinical / professional experience**,** years**	
< 5	1 (4.3)
5–10	2 (8.7)
11–15	5 (21.7)
16–20	2 (8.7)
> 20	13 (56.5)
**Experience with patient-specific subperiosteal implants**	
None	1 (4.3)
Limited (< 10 cases)	5 (21.7)
Moderate (10–50 cases)	4 (17.4)
Extensive (> 50 cases)	12 (52.2)
Not applicable	1 (4.3)
**Research / academic activity**	
None	4 (17.4)
Occasional involvement	6 (26.1)
Active researcher	13 (56.5)
**Publications in the field**	
Yes	15 (65.2)
No	8 (34.8)
**Experience with digital workflows**	
None	0 (0.0)
Basic	0 (0.0)
Intermediate	7 (30.4)
Advanced	16 (69.6)

Data are presented as n (%). Percentages are based on *N* = 23 unless otherwise indicated

*Professional setting was a multiple-response item. One participant selecting “Other” described a combined hospital-based and private-practice setting and was recoded to both stated settings; one response describing previous hospital-based practice was retained as “Other,” so percentages may sum to > 100%

†Geographical regions were derived from the self-reported country/region of practice; one participant did not provide a geographical response

### Delphi round 1

Round 1 evaluated 43 preliminary statements across five predefined thematic domains: Indications; Surgical Workflow; Prosthetic Rehabilitation; Outcomes and Complications; and Follow-up and Maintenance. Thirty statements (69.8%) achieved the predefined consensus threshold, whereas 13 (30.2%) did not achieve consensus (Table 2). The 13 non-consensus statements and two statements that achieved consensus but underwent substantive revision following participant feedback were included in Round 2, resulting in 15 statements for re-evaluation.

**Table 2:** Overview of Delphi statement revisions and consensus outcomes across two rounds

Thematic domain	Round 1 Items, *n*	Round 1 Consensus, *n* (%)	Round 1 No consensus, *n* (%)	Round 2 Items evaluated, *n*	Round 2 Consensus, *n* (%)	Round 2 No consensus, *n*	Structural changes affecting item count	Final consensus statements, *n*
Indications	10	6 (60.0)	4 (40.0)	4	3 (75.0)	1	No change in the number of statements; four non-consensus statements were revised and re-evaluated.	9
Surgical Workflow	8	8 (100.0)	0 (0.0)	0	–	–	No change in the number of statements; all statements achieved consensus in Round 1 and were retained.	8
Prosthetic Rehabilitation	8	6 (75.0)	2 (25.0)	2	2 (100.0)	0	No change in the number of statements; two non-consensus statements were substantially revised and re-evaluated.	8
Outcomes and Complications	9	2 (22.2)	7 (77.8)	7	5 (71.4)	2	No change in the number of statements; seven non-consensus statements were revised and re-evaluated.	7
Follow-up and Maintenance	8	8 (100.0)	0 (0.0)	2	2 (100.0)	0	No change in the number of statements; two consensus statements were refined and re-evaluated following participant feedback.	8
**Total**	**43**	**30 (69.8)**	**13 (30.2)**	**15**	**12 (80.0)**	**3**	**No statements were split**,** consolidated**,** or excluded during Round 2; three statements remained without consensus after Round 2.**	**40**

Note: Consensus was predefined as ≥75% of participants selecting agree or strongly agree. Round 2 included statements that had not achievedconsensus in Round 1, together with consensus statements that underwent substantive refinement following participant feedback. As nostatements were split, consolidated, or excluded during Round 2, the final number of consensus statements reflects the 40 statements achievingconsensus after completion of both Delphi rounds

Consensus varied across the five thematic domains. All eight Surgical Workflow statements and all eight Follow-up and Maintenance statements achieved consensus in Round 1. Six of eight Prosthetic Rehabilitation statements, six of ten Indications statements, and two of nine Outcomes and Complications statements met the predefined consensus threshold.

Revisions between rounds reflected participant comments concerning careful patient selection, the relationship between patient-specific subperiosteal implants and alternative treatment approaches, individualized prosthetic loading and radiographic surveillance, soft-tissue considerations, and the limitations of the available long-term evidence. Fixed numerical estimates and prescriptive wording were removed or qualified where supporting evidence remained heterogeneous. The statement-level wording changes and their rationales are presented in Table 3 and Supplementary Table S2.

**Table 3:** Round 2 evaluation and final disposition of revised Delphi statements

Theme / R2 item	Round 1 result Median (IQR); agreement (%)	Reason for revision	Round 2 result Median (IQR); agreement (%)	Consensus	Final disposition
*Indications* **5. Single-Stage Functional Rehabilitation**	4 (2.75–5); 60.9	Restricted to selected cases where conventional endosseous approaches are not feasible, less predictable, or would require extensive augmentation.	5 (4–5); 95.2	Yes	Retained as final consensus statement
*Indications* **7. Medically Compromised Patients**	4 (3–5); 69.6	Emphasized careful patient selection, medical fitness for surgery, reduction of staged procedures, and use where conventional treatment is not feasible or entails unacceptable surgical risk.	4 (4–5); 85.7	Yes	Retained as final consensus statement
*Indications* **8. Head and Neck Radiotherapy**	3 (2–4); 43.5	Reframed as an individualized risk–benefit assessment considering radiation dose, anatomical site, time since radiotherapy, tissue condition, and comparison with conventional endosseous approaches.	4 (3–5); 71.4	No	No consensus; not included in final consensus set
*Indications* **10. Patient Information**	4 (2.75–5); 69.6	Focused on individualized digital planning, the comparatively less established evidence and protocols, alternative treatments, risks and benefits, and long-term follow-up.	5 (4–5); 85.7	Yes	Consensus achieved; minor editorial refinement
*Prosthetic Rehabilitation* **1. Timing of Prosthetic Rehabilitation**	3.5 (2–4); 47.8	Removed the fixed 2–3-week interval and individualized prosthetic timing according to implant fixation, prosthetic design, soft-tissue conditions, and case complexity.	4 (3–5); 76.2	Yes	Consensus achieved; minor editorial refinement
*Prosthetic Rehabilitation* **2. Immediate Prosthetic Loading**	2.5 (1–4); 43.5	Permitted immediate loading when accurate adaptation and rigid fixation are achieved and recommended revision or re-planning when fit or stability is inadequate.	4 (4–5); 81.0	Yes	Consensus achieved; minor editorial refinement
*Outcomes and Complications* **1. Overall Survival**	4 (3–5); 69.6	Removed specific survival percentages and emphasized favorable short- to medium-term outcomes while acknowledging the need for longer-term, higher-quality evidence.	5 (4–5); 95.2	Yes	Retained as final consensus statement
*Outcomes and Complications* **3. Late Biological Complications (1)**	3.5 (2–4.25); 50.0	Removed fixed complication rates and acknowledged variation according to implant design, surgical technique, follow-up duration, and reporting methodology.	5 (4–5); 100.0	Yes	Retained as final consensus statement
*Outcomes and Complications* **4. Locking Screw Systems**	2.5 (1–3); 52.2	Restricted locking screw use to selected implant designs where implant thickness and anatomical location permit their use and additional fixation stability is desired.	3 (3–4); 47.4	No	No consensus; not included in final consensus set
*Outcomes and Complications* **5. Partial Implant Exposure**	4 (3–5); 73.9	Removed the fixed exposure rate and added implant-, material-, anatomical-, and patient-related qualifications.	5 (4–5); 81.0	Yes	Retained as final consensus statement
*Outcomes and Complications* **6. Mechanical Complications**	4 (3–4); 73.9	Removed the fixed complication rate and described reported mechanical complications as generally infrequent and manageable while acknowledging limited evidence.	4 (3–5); 52.4	No	No consensus; not included in final consensus set
*Outcomes and Complications* **7. Prosthesis Type Comparison**	4 (2.75–4); 65.2	Removed the claim of comparable outcomes and acknowledged high reported survival for both prosthesis types while emphasizing limited direct comparative evidence.	4 (4–5); 90.5	Yes	Retained as final consensus statement
*Outcomes and Complications* **8. Outcomes in Complex Cases**	4 (1.75–5); 56.5	Described favorable outcomes in small case series without claiming consistently high survival or low complication rates and explicitly acknowledged limited, heterogeneous evidence.	5 (4–5); 90.5	Yes	Retained as final consensus statement
*Follow-up and Maintenance* **2. Early Postoperative Monitoring**	4 (3.75–5); 78.3	Refined wording to emphasize that the first postoperative weeks are critical for active detection and management of early complications.	5 (5–5); 90.5	Yes	Retained as final consensus statement
*Follow-up and Maintenance* **4. Radiographic Surveillance**	4 (3.75–5); 78.3	Changed routine periodic surveillance to individualized/selective radiographic evaluation guided by clinical findings while acknowledging limited diagnostic value in some cases.	5 (4–5); 90.5	Yes	Consensus achieved; minor editorial refinement

Note: Round 1 and Round 2 results are reported as median Likert score, interquartile range (IQR), and percentage agreement. Consensus was predefined as ≥ 75% agreement (ratings of 4 or 5). Round 2 evaluated 15 revised statements: 13 that had not achieved consensus in Round 1 and two Round 1 consensus statements that underwent substantive refinement. Twelve of 15 statements (80.0%) achieved consensus in Round 2

### Delphi round 2

Round 2 evaluated 15 revised statements: 13 that had not achieved consensus during Round 1 together with two that had achieved consensus but underwent substantive revision. Of the 15 revised statements evaluated in Round 2, 12 (80.0%) achieved the predefined consensus threshold (Table 3).

Three statements remained below the consensus threshold: the use of patient-specific subperiosteal implants in patients with previous head and neck radiotherapy (15/21, 71.4% agreement), the use of locking screw fixation systems (9/19, 47.4% agreement), and the frequency and manageability of mechanical complications (11/21, 52.4% agreement). These statements were classified as non-consensus statements and were not included in the final consensus set.

### Final consensus statements

Following completion of both Delphi rounds, 40 of the 43 original statements achieved consensus. The final set comprised nine statements on Indications, eight on Surgical Workflow, eight on Prosthetic Rehabilitation, seven on Outcomes and Complications, and eight on Follow-up and Maintenance. Thirty-six statements were retained in the wording in which consensus was achieved, whereas four underwent post-round wording refinement.

The complete set of final consensus statements is presented in Table 4. A detailed statement-by-statement audit trail, including the original and revised statement wording, participant feedback, voting outcomes, and final disposition for each statement across both Delphi rounds, is provided in Supplementary Table S2.

**Table 4:** Final consensus statements for patient-specific subperiosteal implants

Thematic domain	No.	Final consensus statement
**Indications**	1	**Severe Atrophy.** Patient-specific implant-supported prostheses are indicated for patients with severe maxillary or mandibular atrophy in whom conventional endosseous root-form implant placement is not feasible due to insufficient bone volume or unfavorable anatomy
	2	**Advanced Alveolar Ridge Resorption.** Patient-specific implants may be indicated for patients with advanced alveolar ridge resorption who would otherwise require extensive bone augmentation procedures associated with high surgical burden or prolonged treatment time
	3	**History of Failed Implants or Grafting.** Patient-specific implant-supported prostheses may be considered as a treatment option in patients with a history of failed conventional implant therapy or failed bone grafting procedures
	4	**Contraindication to Alternative Graftless Solutions.** Patient-specific implants may be considered when alternative graftless solutions, such as zygomatic or pterygoid implants, are contraindicated, anatomically not feasible, or associated with increased risk
	5	**Single-Stage Functional Rehabilitation.** Single-stage or early functional rehabilitation using patient-specific implant-supported prostheses may be considered in selected patients where conventional endosseous implant approaches are not feasible, less predictable, or would require extensive augmentation procedures
	6	**Complex Anatomical Situations.** Patient-specific implants are indicated for complex anatomical situations that require customized implant geometry to achieve a prosthetically driven rehabilitation
	7	**Medically Compromised Patients.** Patient-specific subperiosteal implant-supported prostheses may be considered in carefully selected medically compromised patients when reducing the number of surgical interventions represents a clinical priority, provided the patient is medically fit for the planned surgical procedure and conventional endosseous implant placement is not feasible or would require extensive augmentation associated with unacceptable surgical risk
	8	**Maxillofacial Reconstruction.** Patient-specific implants are indicated in selected cases of maxillofacial reconstruction following trauma, tumor resection, or congenital defects when conventional reconstructive options are limited
	9	**Patient information.** Patients should be informed that contemporary patient-specific subperiosteal implant treatment involves individualized digital design and surgical planning, and that the available clinical evidence, indications, and treatment protocols are less established and more heterogeneous than those for conventional augmentation procedures, zygomatic implants, or tilted implants. Informed consent should include shared discussion of alternative treatment options, anticipated benefits, potential risks, existing evidence, uncertainties, and the importance of long-term follow-up.
**Surgical Workflow**	1	**Digital Preoperative Planning.** Patient-specific implant-supported prostheses require a fully digital preoperative workflow integrating appropriate three-dimensional imaging, virtual surgical planning, and CAD/CAM-based implant design to ensure accurate anatomical adaptation and prosthetically driven positioning.
	2	**Multidisciplinary Collaboration.** Multidisciplinary collaboration between surgeons, restorative clinicians, and biomedical engineers is a fundamental component of the surgical workflow for patient-specific implants, particularly during planning, design approval, and prosthetic planning
	3	**Prosthetically Driven Virtual Planning.** Preoperative virtual planning should incorporate prosthetically driven principles, including implant emergence profile, implant positioning, and anticipated prosthetic design, prior to manufacturing of the patient-specific implant
	4	**Additive Manufacturing and CAD/CAM Production.** Manufacturing of patient-specific implants typically involves additive manufacturing or CAD/CAM milling using biocompatible materials, with design features tailored to the patient’s anatomy and planned fixation strategy
	5	**Simplified or Single-Stage Surgical Approach.** The surgical placement workflow for patient-specific implants may allow for a single-stage or simplified surgical approach, as implant positioning and geometry are predetermined during the digital planning phase
	6	**Rigid Fixation for Primary Stability.** Rigid fixation of patient-specific implants to the underlying bone using an appropriate number and distribution of screws is a key element of the surgical workflow, particularly for subperiosteal designs, to achieve primary stability and immediate or early functional loading when indicated.
	7	**Intraoperative Accuracy and Plan Transfer.** Accurate intraoperative transfer of the virtual plan to the clinical setting is critical to the surgical workflow, relying on precise implant fit, orientation markers, and predesigned fixation points rather than extensive intraoperative modification
	8	**Postoperative Assessment and Prosthetic Integration.** The surgical workflow for patient-specific implant-supported prostheses should include predefined protocols for postoperative assessment and prosthetic integration, aligned with the initial digital plan to ensure continuity between surgical and restorative phases
**Prosthetic Rehabilitation**	1	**Timing of Prosthetic Rehabilitation.** The timing of prosthetic rehabilitation for patient-specific subperiosteal implants should be individualized according to implant design, extent of fixation, soft-tissue conditions, prosthetic requirements, and case complexity. Immediate or early fixed provisionalization may be considered when accurate implant adaptation, rigid fixation, and appropriate prosthetic stabilization are achieved, while delivery of the definitive prosthesis should be individualized according to soft-tissue maturation and clinical stability
	2	**Immediate Prosthetic Loading.** Immediate prosthetic loading of patient-specific subperiosteal implants may be undertaken when accurate passive implant adaptation, rigid fixation, and appropriate prosthetic stabilization are achieved, using multiple fixation screws and, where indicated, cross-arch provisionalization. An implant with inadequate fit or insufficient fixation should not be loaded and should undergo removal, revision, or re-planning before treatment proceeds
	3	**Passive Fit and Load Distribution.** Prosthetic designs must ensure passive fit and appropriate load distribution to prevent mechanical complications such as abutment fracture, screw loosening, or prosthetic material chipping
	4	**Prosthetic Material Selection.** Prosthetic materials should prioritize rigidity and durability, including acrylic resin, PEEK, or metal-reinforced frameworks, to reduce implant stress and enhance functional longevity
	5	**Soft Tissue Management.** Soft tissue management, including assessment of tissue thickness, keratinized gingiva, and alveolar morphology, is essential to prevent mucosal recession, implant exposure, and peri-implant complications
	6	**Patient-Reported Outcomes.** Patient-reported outcomes should be considered a critical component of prosthetic rehabilitation, as patient-specific implants demonstrate high patient satisfaction, functional recovery, and improvements in oral health-related quality of life
	7	**Occlusal Planning.** Occlusal planning must aim for balanced load distribution across implants to minimize stress concentration and optimize prosthetic longevity
	8	**Digital Prosthetic Workflows.** Digital workflows integrating CAD/CAM, STL-based design, and photogrammetry, and 3D printing should be used to ensure accurate prosthetic fit, reduce intraoperative adaptation, and enhance both functional and esthetic outcomes
**Outcomes and Complications**	1	**Overall Survival.** Digitally manufactured subperiosteal implants have demonstrated favorable short- to medium-term survival outcomes in published studies; however, longer-term follow-up and additional high-quality clinical evidence are still required to confirm long-term predictability and stability
	2	**Early Postoperative Complications.** Early postoperative complications (≤ 14 days), such as pain, swelling, and discomfort, are reported in approximately 25–30% of cases and are generally mild, self-limiting, and associated with minimal impact on implant survival
	3	**Late Biological Complications (1).** Late biological complications associated with patient-specific subperiosteal implants, including infection, soft tissue dehiscence, and chronic discomfort or pain, have been reported in the literature; however, the incidence and severity of these complications appear to vary according to implant design, surgical technique, follow-up duration, and reporting methodology, and further long-term clinical data are required
	4	**Partial Implant Exposure.** Partial implant exposure has been reported in subperiosteal implants and may not necessarily compromise medium-term implant survival or prosthetic function when appropriately managed; however, outcomes may vary according to implant design, material, anatomical location, and patient-related factors.
	5	**Prosthesis Type Comparison.** Both full-arch and bridge prostheses supported by patient-specific subperiosteal implants have demonstrated high survival rates in published studies, generally exceeding 90%, although direct comparisons between prosthesis types remain limited due to heterogeneity in study designs, follow-up periods, and outcome reporting.
	6	**Outcomes in Complex Cases.** Subperiosteal implants have been used in complex clinical scenarios, including revision cases, post-oncological reconstructions, medication-related osteonecrosis of the jaw, and congenital craniofacial anomalies, with reported favorable outcomes in small case series. However, the current evidence base remains limited and heterogeneous, and further larger-scale and long-term studies are required to confirm their predictability in these indications
	7	**Patient-Reported Outcomes.** Patient-reported outcomes, including comfort, function, aesthetics, and oral health-related quality of life, are consistently positive following digitally planned subperiosteal implants, with reported improvements in chewing, speech, lip support, and facial aesthetics
**Follow-up and Maintenance**	1	**Structured Follow-Up Protocols.** Patient-specific implant-supported prostheses should be monitored using structured follow-up protocols, including regular clinical and radiographic assessments, particularly during the first 12 months after placement to detect early biological or mechanical complications
	2	**Early Postoperative Monitoring.** Close postoperative follow-up during the first weeks after surgery is recommended to monitor soft tissue healing, implant stability, and patient comfort, as this period is critical for detecting and managing early postoperative complications
	3	**Soft Tissue and Peri-implant Assessment.** Routine follow-up visits should include assessment of peri-implant soft tissues, including signs of inflammation, bleeding, mucosal recession, or implant exposure, as these parameters are critical indicators of long-term implant success
	4	**Radiographic Surveillance.** Radiographic evaluation should be individualized according to the clinical situation, implant design, reconstruction complexity, and patient-specific risk factors. Baseline postoperative imaging and subsequent radiographic assessment may be considered to evaluate implant position, fixation, and potential pathological bone changes, while additional imaging should be guided by clinical findings and recognition that patient-specific subperiosteal frameworks may limit the diagnostic value of some radiographic examinations.
	5	**Maintenance and Hygiene Protocols.** Long-term maintenance of patient-specific implants requires individualized oral hygiene instructions and scheduled professional maintenance visits to support peri-implant health and minimize biological complications
	6	**Management of Biological Complications.** Biological complications such as soft tissue infections or implant exposure should be addressed promptly through hygiene protocols, antibiotics, or prosthetic adjustments to maintain functional outcomes and prevent implant loss
	7	**Prosthetic Maintenance and Adjustments.** Regular prosthetic evaluation during follow-up is necessary to identify occlusal wear, screw loosening, or minor prosthetic complications, which can usually be managed with simple maintenance or prosthetic adjustments
	8	**Importance of Long-Term Follow-Up.** Long-term follow-up is essential to evaluate implant survival, functional stability, and patient-reported outcomes, as mid- to long-term evidence for digitally manufactured patient-specific implants continues to evolve

Note: Consensus was predefined as ≥75% of participants selecting agree or strongly agree. This table presents the 40 statements that achievedconsensus across the two Delphi rounds. Where minor post-round editorial refinement was indicated, the refined wording is presented. Statements that did not achieve consensus after Round 2 are not included in this table and are documented in Supplementary Table S2 for furtherdiscussion

## Discussion

This modified Delphi study established multidisciplinary expert consensus on 40 statements addressing the clinical application of patient-specific subperiosteal implants across five domains: indications, surgical workflow, prosthetic rehabilitation, outcomes and complications, and follow-up and maintenance. Consensus was strongest for surgical workflow and follow-up and maintenance, for which all initial statements achieved the predefined threshold. Greater uncertainty was evident for indications and clinical outcomes, particularly where recommendations depended on limited comparative or long-term evidence. After two rounds, three issues remained without consensus: the use of patient-specific subperiosteal implants in patients with previous head and neck radiotherapy, the specific role of locking screw fixation, and the frequency of mechanical complications. Although these findings identify unresolved clinical questions, lack of consensus should not necessarily be interpreted as opposition to the underlying clinical concept. Delphi ratings may also be influenced by statement wording, particularly when a statement contains several conditions or combines distinct clinical propositions. This was especially relevant to the mechanical complications statement, which grouped minor prosthetic events with implant mobility and addressed both their frequency and manageability. By contrast, the comments concerning radiotherapy and locking screws revealed more substantive differences in clinical and biomechanical opinion, although the conditional and multicomponent wording of these statements may also have influenced individual ratings.

The overall direction of consensus is consistent with the contemporary literature while remaining appropriately cautious. Recent reports describe favorable outcomes in severe jaw atrophy, revision cases, oncological defects, medication-related osteonecrosis, and other complex situations. However, the available evidence is predominantly observational and remains heterogeneous with respect to implant design, prosthetic configuration, outcome definitions, complication reporting, and duration of follow-up [7, 12]. Current evidence therefore supports clinical feasibility and encouraging early outcomes, but should not be interpreted as establishing equivalence or superiority to established treatment approaches.

### Indications and patient selection

Patient selection emerged as a central theme of the present consensus. The panel supported patient-specific subperiosteal implants primarily for severe maxillary or mandibular atrophy when conventional endosseous implant placement is not feasible, would require extensive augmentation, or has previously failed. This position is consistent with the contemporary literature, in which severe alveolar atrophy and previous reconstructive or implant failure represent the most commonly reported indications [2, 3, 23–25]. The available evidence is most developed for full-arch rehabilitation of the severely atrophic maxilla, whereas sectional, single-tooth, congenital, and oncological applications remain supported by smaller and more heterogenous clinical series [12].

Avoidance of extensive augmentation was also recognized as an important potential advantage. Compared with staged grafting procedures, patient-specific implants may reduce treatment time, donor-site morbidity, and the overall surgical burden in appropriately selected patients [2]. Individual case reports and case series have demonstrated successful rehabilitation of patients in whom conventional augmentation was declined, unsuccessful, or anatomically challenging, including localized posterior maxillary defects and severely resorbed jaws [1, 26, 27]. These reports support clinical feasibility but do not establish superiority over alternative treatment approaches. A recent comparative study reported broadly comparable five-year survival between zygomatic and subperiosteal implants in severe maxillary atrophy, but differences in indications, complications, and treatment strategies preclude a general conclusion of equivalence [28].

The panel also acknowledged a potential role in selected maxillofacial reconstruction cases following trauma, tumor resection, or medication-related osteonecrosis. Customized implant geometry may facilitate integrated skeletal and prosthetic rehabilitation, although the available evidence remains largely limited to case reports and small series [3, 8, 11, 29, 30]. However, the complexity of these cases requires individualized multidisciplinary planning, as virtual planning can support but not replace clinical judgement and intraoperative decision-making.

The greatest uncertainty concerned medically compromised and previously irradiated patients. While consensus was ultimately achieved for carefully selected medically compromised individuals, previous head and neck radiotherapy remained below the predefined consensus threshold after two Delphi rounds. This likely reflects the influence of radiation dose and distribution, tissue quality and vascularity, anatomical site, time since treatment, and overall patient risk. The 71.4% agreement in Round 2 was close to the threshold and should not be interpreted as establishing radiotherapy as a contraindication. Because the revised statement combined several considerations, including flap elevation, periosteal manipulation, radiation dose, anatomical site, time since treatment, and comparison with conventional endosseous treatment, participants may have supported individualized assessment while disagreeing with specific elements. The qualitative comments also indicated genuine uncertainty about whether patient-specific subperiosteal implants reduce reconstructive morbidity or introduce additional risks in irradiated soft tissues. Accordingly, the panel favored cautious, multidisciplinary case-by-case decision-making rather than either general endorsement or exclusion.

### Surgical workflow

Surgical workflow represented the area of strongest agreement in the Delphi process, with all eight statements achieving consensus in Round 1. The panel regarded contemporary patient-specific subperiosteal implant treatment as fundamentally dependent on a digitally integrated pathway combining three-dimensional imaging, virtual planning, CAD/CAM design, prosthetically driven positioning, accurate manufacturing, and controlled transfer of the approved plan to surgery. This emphasis is consistent with the systematic review by Al-Nawas and Bär [7], which identified virtual surgical planning and customization as defining components of contemporary digitally manufactured subperiosteal implants.

Importantly, planning should be prosthetically rather than purely anatomically driven. The position and emergence of transmucosal components, anticipated prosthesis, available fixation sites, and relevant anatomical structures should be considered before implant manufacture. Dimitroulis et al. [4] described combining CT-derived DICOM data with digital information from the existing or planned dentition to determine transmucosal post positions, followed by review and approval of the design by the surgical and restorative teams. Gellrich et al. [5] similarly emphasized prosthodontic backward planning. These observations support the panel’s unanimous view that collaboration between surgeons, restorative clinicians, and biomedical engineers is integral to treatment planning and design approval.

The value of virtual planning ultimately depends on accurate intraoperative transfer. Precise framework adaptation, predetermined fixation points, orientation features, and appropriate surgical exposure are therefore essential [31]. In complex reconstruction, De Riu et al. [32] illustrated how implant design, fixation holes, cutting guides, and prosthetic requirements can be reviewed collaboratively between the engineering and surgical teams before manufacture. Nevertheless, virtual planning cannot eliminate intraoperative uncertainty, particularly when anatomy has changed between imaging and surgery or unexpected tissue conditions are encountered.

Rigid fixation was another consistent feature of the consensus. Modern designs rely on multiple screw fixation to achieve primary stability and maintain intimate framework-to-bone adaptation, distinguishing them biomechanically from many historical subperiosteal implants [5]. Dimitroulis et al. [4] also noted that inadequate imaging quality or prolonged intervals between imaging and implantation may compromise fit because of imaging inaccuracies or subsequent bone remodeling. Thus, although patient-specific implant placement may permit a simplified or single-stage procedure, this depends on substantial precision during the preoperative planning and manufacturing phases. In this context, Goh et al. [6] recommended anchoring the framework to the most stable basal cortical bone, away from the resorbable alveolar crest, and using a sufficient number of osteosynthesis screws to broaden force distribution and limit framework mobility. This recommendation is consistent with biomechanical studies indicating that framework geometry, fixation location, screw diameter and distribution, and anchorage to stable basal bone influence stress transmission and construct stability [33–35]. However, most of this evidence is derived from finite-element or laboratory investigations and cannot establish the clinical superiority of a particular fixation strategy.

The absence of consensus on locking screws should be interpreted in this context. Participant comments reflected a substantive biomechanical disagreement: some considered locking screws advantageous when additional fixation stability is required, whereas others were concerned that locking mechanisms could prevent intimate adaptation, lift the framework from the underlying bone, or introduce internal stresses. The Round 2 statement also combined several selection criteria, implant thickness, anatomical location, implant design, and desire for additional stability, with the proposition that locking screws may be beneficial. Disagreement with any one of these elements may therefore have influenced the rating. The findings do not support routine locking-screw fixation but instead indicate that fixation should be individualized according to framework design, passive fit, bone quality, anatomical location, and the mechanical objective of the construct.

### Prosthetic rehabilitation

Prosthetic rehabilitation was another area of substantial agreement, with six of eight statements reaching consensus in Round 1 and the remaining statements refined before re-evaluation. A central message was that prosthetic planning should be integrated from the outset rather than treated as a secondary phase after implant placement. The position, angulation, and emergence of transmucosal components should reflect the intended restorative outcome while also considering opposing dentition, hygiene access, soft-tissue architecture, and load distribution. Contemporary workflows combining existing prostheses, diagnostic wax-ups, intraoral scans, and CT-derived anatomy reflect this prosthetically driven approach [4, 26, 31, 36, 37].

The timing of loading required greater qualification. Immediate or early provisionalization has been reported in contemporary series, but the panel did not support a rigid universal protocol. Dimitroulis et al. [4] provided immediate fixed provisional prostheses in 15 of 21 cases, whereas Ayhan et al. [38] used both immediate and delayed rehabilitation. More recent reports have also described immediate-load protocols for full-arch rehabilitation, although patient selection and technical protocols remain heterogenous [37]. These differing protocols support the Delphi recommendation that loading should be individualized according to primary stability, implant design, soft-tissue condition, prosthetic configuration, and patient-specific risk.

Passive fit, rigidity, durability, and favorable force distribution were also emphasized. A range of restorative approaches has been reported, including resin- or PMMA-based provisional restorations supported by titanium frameworks and definitive CAD/CAM-fabricated ceramic or zirconia prostheses [4, 26, 36]. PEEK frameworks have also been described, but available comparisons and case series remain too small to demonstrate material superiority [39, 40]. Current evidence does not support the superiority of a specific restorative material or configuration.

Biomechanical considerations remain important because functional loads are transmitted through the transmucosal components to the framework and fixation system. Finite element studies indicate that framework material, cantilever design, wing configuration, thickness, and screw distribution influence stress transmission, although the clinical significance of these models remains uncertain [34, 41]. Clinical reports and evidence syntheses have documented abutment fracture, screw loosening, ceramic chipping, acrylic fracture, and loss of cementation [36, 38, 42]. Overall, the Delphi findings support a prosthetically driven and individualized rehabilitation strategy, with particular emphasis on passive fit, load distribution, and maintainability.

### Outcomes and complications

Outcomes and complications generated substantially greater uncertainty than surgical workflow, with only two of nine statements achieving consensus in Round 1. Subsequent revisions moved several statements away from fixed numerical estimates and categorical claims toward more qualified formulations reflecting heterogeneity in the available evidence. This was particularly evident for implant survival, late biological complications, partial exposure, and comparisons between prosthetic configurations.

The available evidence supports this cautious position. A recent meta-analysis of 11 studies involving 268 patients and 369 implants estimated pooled survival at 97.8% for follow-up periods of up to three years, whereas the longest available six-year cohort reported a survival rate of 54.1%; longer-term evidence was based on considerably fewer observations [13]. Similarly, the scoping review by Vaira et al. [12] found that only approximately ten clinical studies reported follow-up beyond three years and only five extended beyond five years. Accordingly, the panel supported describing current survival outcomes as favorable in the short to medium term without assuming established long-term predictability.

The distinction between implant survival and broader treatment success is particularly important. Clinical case series demonstrate that an implant may remain functional despite soft tissue exposure, previous infection, prosthetic maintenance, or other events that would prevent it from meeting a stricter definition of success. Vatteroni et al. [43], using Stability, Inflammation and Offset criteria, reported 100% cumulative survival at two years but a success rate of 73.1%. Likewise, Onica et al. [14] reported that approximately 97% of implants remained in situ after six years, whereas only 25% of cases fulfilled the study’s success criteria. This demonstrates that implant retention alone may not adequately characterize treatment outcome and supports assessment incorporating biological, mechanical, prosthetic, functional, and patient-centered parameters [43].

Biological complications were a major focus of the Delphi discussion. Early postoperative pain and swelling appear relatively common but are generally transient [7]. Late complications include infection, dehiscence, chronic discomfort, and particularly framework exposure, although their reported incidence varies considerably. Al-Nawas and Bär [7] identified partial exposure in 37% of evaluable cases but noted that it did not appear to compromise short-term implant survival. Anitua et al. [44] similarly reported partial exposure in 25.6% of patients in their systematic review, whereas a multicentre full-arch study reported limited asymptomatic exposure in 9.7% of cases [45]. In contrast, Van den Borre et al. [46] documented substantially higher frequencies of recession or exposure in a selected maxillary cohort and identified thin soft-tissue phenotype and mucositis as relevant risk factors. These differences illustrate the effects of implant design, follow-up duration, anatomical site, and outcome definition on reported complication rates. The panel therefore distinguished partial exposure from implant failure and emphasized that its consequences depend on implant design, anatomical site, soft-tissue conditions, and patient-related factors. Long-term observations further suggest that coupling-element position, framework geometry, proximity to natural teeth, and biofilm accumulation may influence the development and clinical consequences of exposure [15]. These observations are consistent with Goh et al. [6], who identified the transmucosal interface as the principal site of bacterial colonization and framework exposure and emphasized the protective role of keratinized peri-implant mucosa in limiting these biological complications.

Mechanical complications generated the greatest disagreement among panel members. Although published studies have reported prosthetic disconnection, screw-related complications, minor prosthetic fractures, and implant mobility as mechanical events [7], participants considered these outcomes to differ substantially in their clinical significance. Specifically, minor prosthetic complications were generally viewed as manageable through prosthetic maintenance or component replacement, whereas framework mobility was regarded as a major complication that may require implant revision or removal. The failure to achieve consensus probably reflected both genuine uncertainty and the construction of the statement. It combined prosthetic disconnection, minor prosthetic fracture, and implant mobility despite their markedly different clinical consequences, and it simultaneously asserted that these events were both “generally infrequent” and “manageable”. A participant could therefore agree that minor prosthetic events are manageable but disagree that implant mobility is manageable, or agree that complications are infrequent while rejecting their treatment as uniformly straightforward. The results should consequently be interpreted as rejection of this composite proposition rather than as evidence that mechanical complications are necessarily common or generally unmanageable. A recent scoping review of fixed partial patient-specific subperiosteal implant rehabilitations similarly distinguished mechanical maintenance events from biologically or mechanically consequential implant complications [42]. Future studies should report prosthetic maintenance events, fixation-related complications, framework mobility, and implant removal separately.

Patient-reported outcomes provide an important complement to technical endpoints. Van den Borre et al. [47] reported high overall satisfaction and favorable oral health-related quality of life following maxillary rehabilitation, while functional and aesthetic rehabilitation has also been documented in oncological and severely atrophic cases [9, 47]. Standardized patient-reported outcome measures should therefore form part of future evaluations of treatment success.

### Follow-up and maintenance

Follow-up and maintenance represented another area of strong agreement, with all eight initial statements achieving the predefined consensus threshold. The panel supported a structured surveillance strategy extending beyond the immediate postoperative period and incorporating soft-tissue assessment, implant stability, prosthetic function, oral hygiene, and patient-reported outcomes. Particular emphasis was placed on the first postoperative weeks, when soft-tissue healing and early complications require close observation, and on continued monitoring during the first year. This approach is consistent with the risk-based maintenance framework proposed by Dessi and Vaira [48], in which recall intensity is adapted to wound healing, soft-tissue phenotype, exposure, inflammation, hygiene capacity, prosthetic design, and previous complications.

Long-term maintenance should focus particularly on peri-implant soft tissues. Implant exposure, mucosal recession, inflammation, and infection remain among the most relevant biological concerns associated with contemporary subperiosteal implants [7]. Accordingly, routine clinical assessment should include inspection for inflammation, bleeding, recession, exposure, discomfort, and changes in implant or prosthetic stability. The importance of active maintenance is illustrated by Roy et al. [36], who reported resolution of bleeding on probing after recall intervals were shortened from six to three months and professional hygiene measures were implemented. Ayhan et al. [38] similarly emphasized oral hygiene education and professional maintenance in patients developing soft-tissue inflammation or recurrent infection.

Radiographic surveillance required greater qualification. The panel did not support indiscriminate imaging at fixed intervals; rather, assessment should be individualized according to the clinical situation, implant design, reconstruction complexity, and patient-specific risk. Baseline and subsequent imaging may assist in evaluating implant position, fixation, and pathological bone changes, while additional investigations should be guided by clinical findings. Published studies have used panoramic and CBCT imaging alongside clinical examination to evaluate implant position, fixation, framework offset, and bone remodelling, but no standardized radiographic surveillance schedule has been validated [43, 49, 50].

Prosthetic surveillance is equally important because maintenance requirements may emerge well after initial healing. Reported events include screw-retained complications, prosthetic instability, loss of cementation, veneering-material fracture, and fracture of provisional restorations [42]. Such events reinforce the panel’s recommendation for regular assessment of occlusion, prosthetic integrity, fixation, and component stability, allowing manageable complications to be addressed before they threaten the implant framework or rehabilitation.

Follow-up should extend beyond implant survival. Patient comfort, function, aesthetics, and oral health-related quality of life are important measures of treatment benefit, with favorable patient-reported outcomes documented after rehabilitation of both severely atrophic and post-oncological cases [47, 51]. Structured long-term follow-up incorporating biological, mechanical, prosthetic, and patient-reported outcomes is therefore important for both patient care and evaluation of this evolving treatment modality.

### Strengths and limitations

Several methodological features strengthen the credibility of the present consensus. The study used a predefined two-round modified Delphi process based on established methodological guidance, including the recommendations of Diamond et al. [17] and the ACCORD reporting guideline [18]. Consensus was defined a priori as at least 75% agreement and applied consistently across both rounds, reducing the risk of post-hoc interpretation of agreement. Anonymous voting, structured feedback, and free-text comments supported independent expert judgement and allowed uncertainty to be considered alongside quantitative agreement.

A further strength was the clinical experience of the panel. More than half of participants had over 20 years of professional experience, 52.2% reported experience with more than 50 patient-specific subperiosteal implant cases, and 69.6% reported advanced experience with digital workflows. In addition, 56.5% were active researchers and 65.2% had published in the field. The combination of quantitative ratings and qualitative feedback also allowed statements to be refined rather than simply maximizing consensus. Three statements remained below the predefined threshold after Round 2 and were therefore excluded from the final consensus set. High retention between rounds (22/23 participants; 95.7%) and the detailed statement-level audit trail in Supplementary Table S2 further support the transparency of the process.

Several limitations nevertheless warrant consideration. Delphi consensus represents the collective judgement of a selected expert panel and should not be regarded as equivalent to evidence from prospective comparative studies or formal clinical practice guidelines. This is particularly important for patient-specific subperiosteal implants because the available literature remains dominated by observational studies, case series, and case reports, with substantial heterogeneity in implant design, indications, outcome definitions, and follow-up duration [7, 12]. Moreover, only 23 of 58 invited experts completed Round 1 (39.7%), so self-selection and non-response bias cannot be excluded. The resulting statements should therefore be interpreted as evidence-informed expert guidance for an evolving treatment modality rather than proof of comparative effectiveness.

The panel was also predominatly composed of oral and maxillofacial surgeons (65.2%) and European participants (73.9%), while prosthodontists and periodontists were represented by only one participant each. These characteristics may limit the generalizability of the findings. Patients were not directly involved, and the preliminary literature search was not a formal systematic review or evidence-grading process.

Finally, some statements remained multicomponent despite revision, particularly those concerning mechanical complications and, to a lesser extent, radiotherapy and locking screws. Because participants rated each statement as a whole, the study cannot determine which individual clause accounted for disagreement. These findings should therefore be interpreted together with the qualitative comments and not solely on the basis of aggregate agreement percentages. The consensus should be revisited as larger prospective studies, standardized outcome reporting, and longer-term evidence become available.

## Conclusions

This modified Delphi study established international multidisciplinary expert consensus on the clinical application of patient-specific subperiosteal implants, resulting in 40 consensus statements spanning indications, surgical workflow, prosthetic rehabilitation, outcomes and complications, and follow-up. Collectively, these statements provide an evidence-informed framework to support patient selection, treatment planning, surgical and prosthetic decision-making, and long-term maintenance in contemporary clinical practice. The consensus positions patient-specific subperiosteal implants as a selective treatment option for carefully selected patients rather than as a replacement for conventional endosseous implant therapy or established reconstructive procedures. At the same time, the panel recognized important areas of uncertainty, particularly regarding management of irradiated patients, locking screw fixation, and mechanical complications, underscoring the need for individualized clinical judgment. As higher-quality comparative studies and longer-term outcome data become available, these consensus recommendations should be refined and validated to support future evidence-based clinical practice guidelines.

Supplementary Table S1: Databases and search terms.

Supplementary Table S2: Summary of Delphi statement revisions and consensus outcomes following twoDelphi rounds.

## Electronic Supplementary Material

Below is the link to the electronic supplementary material.

Supplementary Table S1 Databases and search terms

Supplementary Table S2 Summary of Delphi statement revisions and consensus outcomes following two Delphi rounds

## Data Availability

The datasets used and analysed during the current study are available from the corresponding author on reasonable request.

## Abbreviations

ACCORD: Accurate consensus reporting document
CAD/CAM: Computer-aided design and computer-aided manufacturing
CBCT: Cone-beam computed tomography
CENTRAL: Cochrane central register of controlled trials
COMET: Core outcome measures in effectiveness trials
CT: Computed tomography
DICOM: Digital imaging and communications in medicine
EMBASE: Excerpta medica database
IQR: Interquartile range
MEDLINE: Medical literature analysis and retrieval system online
PEEK: Polyetheretherketone
PMMA: Polymethyl methacrylate
SPSS: Statistical package for the social sciences
