# Supplementary material for: International multidisciplinary expert consensus on the clinical application of patient-specific subperiosteal implants: a modified Delphi study
Source: Int J Implant Dent. 2026 Oct 26;12:56. doi: 10.1186/s40729-026-00729-8 (PMC13710477; doi:10.1186/s40729-026-00729-8)
Supplement: Supplementary file 1 [file 40729_2026_729_MOESM1_ESM.docx]

**Supplementary Table S1**	Databases and search terms

| **Databases** | **Keywords** |
| --- | --- |
| **Published studies**  **PubMed**  **(2000 - December 19, 2025)**  Theme 1:  Indications  Theme 2:  Surgical Workflow  Theme 3:  Prosthetic Rehabilitation  Theme 4:  Outcomes and Complications  Theme 5:  Follow-up and Maintenance  **EMBASE via Ovid**  **(2000 - December 19, 2025)**  Theme 1:  Indications  Theme 2:  Surgical Workflow  Theme 3:  Prosthetic Rehabilitation  Theme 4:  Outcomes and Complications  Theme 5:  Follow-up and Maintenance  **Cochrane Central Register of Controlled Trials (CENTRAL) via Ovid**  **(December 19, 2025)**  Theme 1:  Indications  Theme 2:  Surgical Workflow  Theme 3:  Prosthetic Rehabilitation  Theme 4:  Outcomes and Complications  Theme 5:  Follow-up and Maintenance | ("Subperiosteal Implants"[MeSH Terms]  OR "Patient-Specific Implants"[Title/Abstract]  OR "3D Subperiosteal Implant"[Title/Abstract]  OR "3D Dental Implants"[Title/Abstract]  OR "Custom-made Subperiosteal Implant"[Title/Abstract])  AND  ("Indications"[Title/Abstract]  OR "Patient Selection"[Title/Abstract]  OR "Treatment Planning"[Title/Abstract]  OR "Atrophic Jaw"[Title/Abstract]  OR "Atrophic Maxilla"[Title/Abstract])  ("Subperiosteal Implants"[MeSH Terms]  OR "Patient-Specific Implants"[Title/Abstract]  OR "3D Subperiosteal Implant"[Title/Abstract]  OR "Custom-made Subperiosteal Implant"[Title/Abstract])  AND  ("Surgical Workflow"[Title/Abstract]  OR "Digital Planning"[Title/Abstract]  OR "CAD/CAM"[Title/Abstract]  OR "Computer Guided Surgery"[Title/Abstract]  OR "Surgical Guide"[Title/Abstract])  ("Subperiosteal Implants"[MeSH Terms]  OR "Patient-Specific Implants"[Title/Abstract]  OR "3D Subperiosteal Implant"[Title/Abstract]  OR "Custom-made Subperiosteal Implant"[Title/Abstract])  AND  ("Prosthetic"[Title/Abstract]  OR "Restorative"[Title/Abstract]  OR "Prosthesis Design"[Title/Abstract]  OR "Immediate Loading"[Title/Abstract]  OR "Delayed Loading"[Title/Abstract])  ("Subperiosteal Implants"[MeSH Terms]  OR "Patient-Specific Implants"[Title/Abstract]  OR "3D Subperiosteal Implant"[Title/Abstract]  OR "Custom-made Subperiosteal Implant"[Title/Abstract])  AND  ("Outcome"[Title/Abstract]  OR "Success Rate"[Title/Abstract]  OR "Survival Rate"[Title/Abstract]  OR "Complication"[Title/Abstract]  OR "Failure"[Title/Abstract]  OR "Prosthetic Complication"[Title/Abstract])  ("Subperiosteal Implants"[MeSH Terms]  OR "Patient-Specific Implants"[Title/Abstract]  OR "3D Subperiosteal Implant"[Title/Abstract]  OR "Custom-made Subperiosteal Implant"[Title/Abstract])  AND  ("Follow-up"[Title/Abstract]  OR "Maintenance"[Title/Abstract]  OR "Monitoring"[Title/Abstract]  OR "Revision"[Title/Abstract]  OR "Patient-Reported Outcomes"[Title/Abstract]  OR "Quality of Life"[Title/Abstract])  'patient specific implant'/exp OR 'subperiosteal implant'/exp OR '3d printed subperiosteal implant' OR 'custom made subperiosteal implant'  AND  'indication' OR 'patient selection' OR 'treatment planning' OR 'atrophic jaw' OR 'atrophic maxilla'  'patient specific implant'/exp OR 'subperiosteal implant'/exp OR '3d printed subperiosteal implant'  AND  'surgical workflow' OR 'digital planning' OR 'cad/cam' OR 'computer guided surgery' OR 'surgical guide'  'patient specific implant'/exp OR 'subperiosteal implant'/exp  AND  'prosthetic' OR 'restorative' OR 'prosthesis design' OR 'immediate loading' OR 'delayed loading'  'patient specific implant'/exp OR 'subperiosteal implant'/exp  AND  'outcome' OR 'success rate' OR 'survival rate' OR 'complication' OR 'failure' OR 'prosthetic complication'  'patient specific implant'/exp OR 'subperiosteal implant'/exp  AND  'follow-up' OR 'maintenance' OR 'monitoring' OR 'revision' OR 'hygiene' OR 'patient reported outcomes' OR 'quality of life'  "subperiosteal implant" OR "patient-specific implant" OR "3D-printed subperiosteal implant" OR "custom-made subperiosteal implant"  AND  “indication” OR "patient selection" OR "treatment planning" OR "atrophic jaw" OR "atrophic maxilla"  "subperiosteal implant" OR "patient-specific implant" OR "3D-printed subperiosteal implant"  AND  "surgical workflow" OR "digital planning" OR CAD/CAM OR "computer-guided surgery" OR "surgical guide"  "subperiosteal implant" OR "patient-specific implant"  AND  prosthetic OR restorative OR "prosthesis design" OR "immediate loading" OR "delayed loading"  "subperiosteal implant" OR "patient-specific implant"  AND  outcome OR "success rate" OR "survival rate" OR complication OR failure OR "prosthetic complication"  "subperiosteal implant" OR "patient-specific implant"  AND  "follow-up" OR maintenance OR monitoring OR revision OR hygiene OR "patient-reported outcomes" OR "quality of life" |
| **Unpublished studies**  **ClinicalTrials.gov**  **(December 19, 2025)**  **ProQuest Dissertation and Theses Global**  **(December 19, 2025)** | ("subperiosteal implant" OR "patient-specific implant" OR "3D printed subperiosteal implant")  AND  ("indication" OR "patient selection" OR "treatment planning" OR "atrophic jaw" OR "atrophic maxilla"  OR "surgical workflow" OR "digital planning" OR "CAD/CAM" OR "computer-guided surgery" OR "surgical guide" OR "prosthetic" OR "restorative" OR "prosthesis design" OR "immediate loading" OR "delayed loading" OR "outcome" OR "success rate" OR "survival rate" OR "complication" OR "failure" OR "prosthetic complication" OR "follow-up" OR "maintenance" OR "monitoring" OR "revision" OR "patient-reported outcomes" OR "quality of life") |
