# Supplementary material for: International multidisciplinary expert consensus on the clinical application of patient-specific subperiosteal implants: a modified Delphi study
Source: Int J Implant Dent. 2026 Oct 26;12:56. doi: 10.1186/s40729-026-00729-8 (PMC13710477; doi:10.1186/s40729-026-00729-8)
Supplement: Supplementary file 2 [file 40729_2026_729_MOESM2_ESM.docx]

Supplementary Table S2

Summary of Delphi statement revisions and consensus outcomes following two Delphi rounds

**Final decision color key**

| **Retained** | Retained as final consensus statement |
| --- | --- |
| **Minor editorial refinement** | Consensus achieved; minor wording refinement recommended |
| **Discuss** | Requires discussion because consensus was not achieved or substantive uncertainty remains |

Theme 1: Indications

| **Original statement** | **Round 1 results**  **Median (IQR); Agreement (%)** | **Modification after round 1** | **Statement presented in round 2** | **Round 2 results**  **Median (IQR); Agreement (%)** | **Consensus** | **Round 2 comments** | **Final decision (retained, minor editorial refinement, discuss, exclude)** |
| --- | --- | --- | --- | --- | --- | --- | --- |
| **1. Severe Atrophy**  Patient-specific implant-supported prostheses are indicated for patients with severe maxillary or mandibular atrophy in whom conventional endosseous root-form implant placement is not feasible due to insufficient bone volume or unfavorable anatomy | 5 (4-5); 87.0 | Not revised | Not presented in round 2 | - | Yes (round 1) | - | **Retained as final consensus statement** |
| **2. Advanced Alveolar Ridge Resorption**  Patient-specific implants may be indicated for patients with advanced alveolar ridge resorption who would otherwise require extensive bone augmentation procedures associated with high surgical burden or prolonged treatment time | 5 (4-5); 95.7 | Not revised | Not presented in round 2 | - | Yes (round 1) | - | **Retained as final consensus statement** |
| **3. History of Failed Implants or Grafting**  Patient-specific implant-supported prostheses are indicated in patients with a history of failed conventional implant therapy or failed bone grafting procedures | 4 (4-5); 91.3 | Not revised | Not presented in round 2 | - | Yes (round 1) | - | **Minor editorial refinement.**  **Reason:** Consensus was achieved in Round 1 (91.3% agreement); however, participant feedback suggested less prescriptive wording and indicated that a history of failed conventional implant therapy or grafting should not, in isolation, be interpreted as an absolute indication.  **Suggested refined statement**  Patient-specific implant-supported prostheses may be considered as a treatment option in patients with a history of failed conventional implant therapy or failed bone grafting procedures |
| **4. Contraindication to Alternative Graftless Solutions**  Patient-specific implants may be considered when alternative graftless solutions, such as zygomatic or pterygoid implants, are contraindicated, anatomically not feasible, or associated with increased risk | 5 (4-5); 91.3 | Not revised | Not presented in round 2 | - | Yes (round 1) | - | **Retained as final consensus statement** |
| **5. Single-Stage Functional Rehabilitation**  Patient-specific implant-supported prostheses may be indicated in patients for whom a single-stage surgical approach with immediate or early functional rehabilitation is clinically desirable | 4 (2.75-5); 60.9 | Revised to restrict the indication to selected cases in which conventional endosseous approaches are not feasible, are less predictable, or would require extensive augmentation | **5. Single-Stage Functional Rehabilitation** Single-stage or early functional rehabilitation using patient-specific implant-supported prostheses may be considered in selected patients where conventional endosseous implant approaches are not feasible, less predictable, or would require extensive augmentation procedures | 5 (4-5) ; 95.2 | Yes (round 2) | Participants supported single-stage or early functional rehabilitation when accurate digital planning, passive implant fit, rigid fixation, adequate prosthetic support, and suitable hard- and soft-tissue conditions are present. Immediate or next-day fixed provisional rehabilitation was considered an important potential advantage. However, staged treatment may remain appropriate when extractions, cystic or osseous defects, substantial bone regularization, or soft-tissue optimization are required | **Retained as final consensus statement** |
| **6. Complex Anatomical Situations**  Patient-specific implants are indicated for complex anatomical situations that require customized implant geometry to achieve a prosthetically driven rehabilitation | 5 (4-5); 87.0 | Not revised | Not presented in round 2 | - | Yes (round 1) | - | **Retained as final consensus statement** |
| **7. Medically Compromised Patients**  Patient-specific implant-supported prostheses may be considered in medically compromised patients when minimizing the number of surgical interventions is a clinical priority | 4 (3-5); 69.6 | Revised to emphasize careful patient selection, medical fitness for surgery, reduction of staged procedures, and use only where conventional endosseous treatment is not feasible or would involve unacceptable surgical risk | **7. Medically Compromised Patients** Patient-specific subperiosteal implant-supported prostheses may be considered in carefully selected medically compromised patients when reducing the number of surgical interventions represents a clinical priority, provided the patient is medically fit for the planned surgical procedure and conventional endosseous implant placement is not feasible or would require extensive augmentation associated with unacceptable surgical risk | 4 (4-5); 85.7 | Yes (round 2) | Participants acknowledged that reducing multiple grafting and staged surgical procedures may benefit carefully selected medically compromised patients who are fit for surgery. However, they emphasized increased risks of impaired soft-tissue healing, dehiscence, infection, and treatment failure in this population. Some considered the approach a possible medium-term solution, and careful individualized risk assessment remains essential | **Retained as final consensus statement** |
| **8. Head and Neck Radiotherapy**  Patient-specific subperiosteal implants require increased surgical intervention and increased periosteal stripping. For example, following radiotherapy, stripping the periosteum to achieve fixation of the patient-specific implant may be associated with considerable risk. | 3 (2-4); 43.5 | Substantially reframed from a predominantly cautionary statement to an individualized risk–benefit assessment considering radiation dose, anatomical site, time elapsed since radiotherapy, tissue condition, and comparison with conventional endosseous approaches. | **8. Head and Neck Radiotherapy**  In patients with a history of head and neck radiotherapy, the use of patient-specific subperiosteal implants requires careful surgical planning, as flap elevation and periosteal manipulation may increase the risk of complications depending on radiation dose, anatomical site, and time elapsed since radiotherapy. Potential advantages and risks should be evaluated on an individual patient basis in comparison with conventional endosseous implant approaches | 4 (3-5); 71.4 | No (round 2) | Participants agreed that evidence remains limited and that irradiated patients require cautious, individualized assessment based on radiation dose and distribution, tissue quality, vascularity, anatomical site, time since treatment, and overall medical condition. Opinions remained divided regarding comparative risk: some emphasized the hazards of extensive flap elevation and compromised soft tissues, whereas others suggested that customized design and strategic fixation may reduce periosteal disruption, extensive grafting, and the morbidity associated with conventional endosseous approaches. | **Discuss — no consensus achieved after Round 2.**  **Reason:** Agreement remained below the predefined 75% threshold, and the comments reveal genuine clinical uncertainty rather than a simple wording problem.  **Potential wording for consensus-meeting discussion**  In patients with a history of head and neck radiotherapy, patient-specific subperiosteal implants should be considered only following individualized multidisciplinary assessment of radiation dose and distribution, anatomical site, tissue vascularity and quality, time elapsed since radiotherapy, and the risks and benefits of alternative reconstructive approaches. Current evidence is insufficient to determine whether they confer lower or higher overall risk than conventional endosseous implant treatment |
| **9. Maxillofacial Reconstruction**  Patient-specific implants are indicated in selected cases of maxillofacial reconstruction following trauma, tumor resection, or congenital defects when conventional reconstructive options are limited | 5 (4-5); 91.3 | Not revised | Not presented in round 2 | - | Yes (round 1) | - | **Retained as final consensus statement** |
| **10. Patient information**  Patients must be informed prior to treatment that patient-specific implants are still an evolving treatment method and have less documentation than other existing methods, such as augmentation, zygomatic implants, and tilted implants | 4 (2.75-5); 69.6 | Revised to focus on individualized digital design and planning, the comparatively less standardized evidence base and clinical protocols, discussion of alternative treatments, risks and benefits, and the importance of long-term follow-up. | **10. Patient information**  Patients should be informed that contemporary patient-specific subperiosteal implant approaches involve individualized digital design and surgical planning, and that current evidence and clinical protocols remain less standardized than those for more established treatment approaches such as conventional augmentation procedures, zygomatic implants, or tilted implants. Informed consent should include discussion of available alternative treatment options, potential risks, benefits, and existing clinical evidence, and importance of long-term follow-up. | 5 (4-5); 85.7 | Yes (round 2) | Participants emphasized realistic expectation-setting, comprehensive informed consent, shared decision-making, discussion of alternative treatments, and the importance of long-term follow-up. They supported acknowledging the comparatively limited clinical evidence but noted that contemporary digital planning and manufacturing workflows may be highly standardized when undertaken by experienced teams. The need for minimum design and clinical protocol standards was also highlighted. | **Minor editorial refinement.**  **Reason:** Consensus was achieved, but the current wording could be interpreted as suggesting that digital design and manufacturing themselves are inadequately standardized. The comments distinguish between **limited or heterogeneous clinical evidence and protocols** and **potentially well-standardized digital manufacturing workflows**.  **Suggested refined statement**  Patients should be informed that contemporary patient-specific subperiosteal implant treatment involves individualized digital design and surgical planning, and that the available clinical evidence, indications, and treatment protocols are less established and more heterogeneous than those for conventional augmentation procedures, zygomatic implants, or tilted implants. Informed consent should include shared discussion of alternative treatment options, anticipated benefits, potential risks, existing evidence, uncertainties, and the importance of long-term follow-up. |

Theme 2: Surgical Workflow

| **Original statement** | **Round 1 results**  **Median (IQR); Agreement (%)** | **Modification after round 1** | **Statement presented in round 2** | **Round 2 results**  **Median (IQR); Agreement (%)** | **Consensus** | **Round 2 comments** | **Final decision (retained, minor editorial refinement, discuss, exclude)** |
| --- | --- | --- | --- | --- | --- | --- | --- |
| **1. Digital Preoperative Planning**  Patient-specific implant-supported  prostheses require a fully digital preoperative workflow integrating three-dimensional imaging (CBCT), virtual surgical planning, and CAD/CAM-  based implant design to ensure accurate anatomical adaptation and prosthetically driven positioning | 5 (5-5); 91.3 | Not revised | Not presented in round 2 | - | Yes (round 1) | - | **Minor editorial refinement.**  **Reason:** Consensus was achieved n Round 1 (91.3% agreement). The specific reference to CBCT was replaced with “appropriate three-dimensional imaging” to clarify that the imaging modality should be selected according to the clinical indication rather than interpreted as requiring CBCT in all cases. This refinement preserves the underlying consensus regarding the integration of three-dimensional imaging within the digital preoperative workflow.  **Suggested refined statement**  Patient-specific implant-supported prostheses require a fully digital preoperative workflow integrating appropriate three-dimensional imaging, virtual surgical planning, and CAD/CAM-based implant design to ensure accurate anatomical adaptation and prosthetically driven positioning. |
| **2. Multidisciplinary Collaboration**  Multidisciplinary collaboration between  surgeons, restorative clinicians, and biomedical engineers is a fundamental component of the surgical workflow for patient-specific implants, particularly during planning, design approval, and prosthetic planning | 5 (4.75-5); 100.0 | Not revised | Not presented in round 2 | - | Yes (round 1) | - | **Retained as final consensus statement** |
| **3. Prosthetically Driven Virtual Planning**  Preoperative virtual planning should incorporate prosthetically driven principles, including implant emergence profile, implant positioning, and anticipated prosthetic design, prior to manufacturing of the patient-specific implant | 5 (4-5); 100.0 | Not revised | Not presented in round 2 | - | Yes (round 1) | - | **Retained as final consensus statement** |
| **4. Additive Manufacturing and CAD/CAM Production**  Manufacturing of patient-specific implants typically involves additive manufacturing or CAD/CAM milling using biocompatible materials, with design features tailored to the patient’s anatomy and planned fixation strategy | 5 (4-5); 87.0 | Not revised | Not presented in round 2 | - | Yes (round 1) | - | **Retained as final consensus statement** |
| **5. Simplified or Single-Stage Surgical Approach**  The surgical placement workflow for patient-specific implants may allow for a single-stage or simplified surgical approach, as implant positioning and geometry are predetermined during the digital planning phase | 5 (4-5); 95.7 | Not revised | Not presented in round 2 | - | Yes (round 1) | - | **Retained as final consensus statement** |
| **6. Rigid Fixation for Primary Stability**  Rigid fixation of patient-specific implants to the underlying bone using multiple screws is a key element of the surgical workflow, particularly for subperiosteal designs, to achieve primary stability and immediate or early functional loading when indicated | 5 (4.75-5); 100.0 | Not revised | Not presented in round 2 | - | Yes (round 1) | - | **Minor editorial refinement.**  **Reason:** Consensus was achieved in Round 1 (100% agreement). The phrase “multiple screws” was refined to “an appropriate number and distribution of screws” to clarify that rigid fixation depends on the fixation strategy rather than screw number alone. This refinement preserves the underlying consensus that rigid fixation is required to achieve primary stability.  **Suggested refined statement**  Rigid fixation of patient-specific implants to the underlying bone using an appropriate number and distribution of screws is a key element of the surgical workflow, particularly for subperiosteal designs, to achieve primary stability and immediate or early functional loading when indicated. |
| **7. Intraoperative Accuracy and Plan Transfer**  Accurate intraoperative transfer of the virtual plan to the clinical setting is critical to the surgical workflow, relying on precise implant fit, orientation markers, and predesigned fixation points rather than extensive intraoperative modification | 5 (4-5); 95.7 | Not revised | Not presented in round 2 | - | Yes (round 1) | - | **Retained as final consensus statement** |
| **8. Postoperative Assessment and Prosthetic Integration**  The surgical workflow for patient-specific implant-supported prostheses should include predefined protocols for postoperative assessment and prosthetic integration, aligned with the initial digital plan to ensure continuity between surgical and restorative phases | 5 (4-5); 91.3 | Not revised | Not presented in round 2 | - | Yes (round 1) | - | **Retained as final consensus statement** |

Theme 3: Prosthetic Rehabilitation

| **Original statement** | **Round 1 results**  **Median (IQR); Agreement (%)** | **Modification after round 1** | **Statement presented in round 2** | **Round 2 results**  **Median (IQR); Agreement (%)** | **Consensus** | **Round 2 comments** | **Final decision (retained, minor editorial refinement, discuss, exclude)** |
| --- | --- | --- | --- | --- | --- | --- | --- |
| **1. Timing of Prosthetic Rehabilitation**  Prosthetic rehabilitation should ideally follow soft tissue healing (approximately 2–3 weeks) in complex  or atrophic cases to reduce biological complications and optimize long-term implant survival | 3.5 (2-4); 47.8 | Substantially revised to remove the fixed 2–3-week interval and individualize prosthetic timing according to implant fixation, prosthetic design, soft-tissue conditions, and case complexity | **1. Timing of Prosthetic Rehabilitation** The timing of prosthetic rehabilitation for patient-specific subperiosteal implants should be individualized according to surgical stability, prosthetic design, soft tissue conditions, and case complexity. Immediate or early provisionalization may be feasible in selected cases, while definitive prosthetic rehabilitation may be delayed until adequate soft tissue healing and clinical stability are achieved | 4 (3-5); 76.2 | Yes (round 2) | Participants generally supported individualized timing but several questioned the term “surgical stability,” suggesting that adequate primary fixation and accurate implant fit should be prerequisites rather than variables determining timing. Many considered immediate fixed provisional rehabilitation the usual or preferred protocol when precise planning, passive fit, rigid fixation, and appropriate prosthetic stabilization are achieved. Definitive prosthesis timing was viewed as more appropriately individualized according to soft-tissue maturation and prosthetic considerations | **Minor editorial refinement**  Reason  Consensus was achieved, but narrowly. The comments reveal that participants largely distinguished between:   - **immediate provisionalization**, often viewed as routine when fit and fixation are satisfactory; and - **definitive prosthetic rehabilitation**, which should remain individualized.   The phrase **“according to surgical stability”** was also questioned because satisfactory fit and fixation should already exist before accepting the implant.  Possible refined wording  **The timing of prosthetic rehabilitation for patient-specific subperiosteal implants should be individualized according to implant design, extent of fixation, soft-tissue conditions, prosthetic requirements, and case complexity. Immediate or early fixed provisionalization may be considered when accurate implant adaptation, rigid fixation, and appropriate prosthetic stabilization are achieved, while delivery of the definitive prosthesis should be individualized according to soft-tissue maturation and clinical stability** |
| **2. Immediate Prosthetic Loading**  Immediate prosthetic loading may be  considered only in cases with sufficient primary implant stability, taking  into account occlusal forces, bone quality, and implant design | 2.5 (1-4); 43.5 | Substantially revised to permit immediate loading when accurate adaptation and rigid fixation are achieved, and to recommend implant revision or re-planning when fit or stability is inadequate | **2. Immediate Prosthetic Loading** Immediate prosthetic loading of patient-specific subperiosteal implants may be considered when accurate implant adaptation and rigid fixation are achieved, with stability provided by multiple fixation screws and, where appropriate, cross-arch provisionalization. In cases of inadequate fit or insufficient primary stability, implant revision or re-planning should be considered before loading | 4 (4-5); 81.0 | Yes (round 2) | Participants generally supported immediate loading when accurate passive adaptation, rigid multi-screw fixation, and appropriate prosthetic or cross-arch stabilization are achieved. Several considered immediate fixed loading the standard protocol rather than an optional approach. There was strong agreement that an implant with inadequate fit or fixation should not be loaded or left in place and should instead be removed, redesigned, or re-planned. One participant noted that press-fit adaptation may reduce the number of screws required in selected implant designs | **Minor editorial refinement**  Reason  The statement achieved consensus, but the comments consistently suggest two refinements:   1. **Passive fit or accurate adaptation should be explicitly verified.** 2. Inadequate fit or fixation should lead to **removal/revision or re-planning**, rather than merely saying this “should be considered.”   Possible refined wording  **Immediate prosthetic loading of patient-specific subperiosteal implants may be undertaken when accurate passive implant adaptation, rigid fixation, and appropriate prosthetic stabilization are achieved, using multiple fixation screws and, where indicated, cross-arch provisionalization. An implant with inadequate fit or insufficient fixation should not be loaded and should undergo removal, revision, or re-planning before treatment proceeds** |
| **3. Passive Fit and Load Distribution**  Prosthetic designs must ensure passive fit and appropriate load distribution to prevent mechanical  complications such as abutment fracture, screw loosening, or prosthetic material chipping | 5 (4-5); 100.0 | Not revised | Not presented in round 2 | - | Yes (round 1) | - | **Retained as final consensus statement** |
| **4. Prosthetic Material Selection**  Prosthetic materials should prioritize  rigidity and durability, including acrylic resin, PEEK, or metal-reinforced  frameworks, to reduce implant stress and enhance functional longevity | 4.5 (3.75-5); 78.3 | Not revised | Not presented in round 2 | - | Yes (round 1) | - | **Retained as final consensus statement** |
| **5. Soft Tissue Management**  Soft tissue management, including  assessment of tissue thickness, keratinized gingiva, and alveolar  morphology, is essential to prevent mucosal recession, implant  exposure, and peri-implant complications | 5 (4-5); 91.3 | Not revised | Not presented in round 2 | - | Yes (round 1) | - | **Retained as final consensus statement** |
| **6. Patient-Reported Outcomes**  Patient-reported outcomes should be  considered a critical component of prosthetic rehabilitation, as patient-  specific implants demonstrate high patient satisfaction, functional  recovery, and improvements in oral health-related quality of life | 5 (4-5); 95.7 | Not revised | Not presented in round 2 | - | Yes (round 1) | - | **Retained as final consensus statement** |
| **7. Occlusal Planning**  Occlusal planning must aim for balanced load  distribution across implants to minimize stress concentration and optimize prosthetic longevity | 5 (4-5); 91.3 | Not revised | Not presented in round 2 | - | Yes (round 1) | - | **Retained as final consensus statement** |
| **10. Digital Prosthetic Workflows**  Digital workflows integrating CAD/CAM,  STL-based design, and photogrammetry, and 3D printing should be used  to ensure accurate prosthetic fit, reduce intraoperative adaptation, and  enhance both functional and esthetic outcomes | 4 (4-5); 87.0 | Not revised | Not presented in round 2 | - | Yes (round 1) | - | **Retained as final consensus statement** |

Theme 4: Outcomes and Complications

| **Original statement** | **Round 1 results**  **Median (IQR); Agreement (%)** | **Modification after round 1** | **Statement presented in round 2** | **Round 2 results**  **Median (IQR); Agreement (%)** | **Consensus** | **Round 2 comments** | **Final decision (retained, minor editorial refinement, discuss, exclude)** |
| --- | --- | --- | --- | --- | --- | --- | --- |
| **1. Overall Survival**  Digitally manufactured subperiosteal implants demonstrate high overall survival rates, ranging from 93.8% to 100%, with a mean survival of approximately 97.6% over follow-up periods extending up to 57 months | 4 (3-5); 69.6 | Substantially revised to remove specific survival percentages and emphasize favorable short- to medium-term outcomes while acknowledging the need for longer-term, higher-quality evidence | **1. Overall Survival**  Digitally manufactured subperiosteal implants have demonstrated favorable short- to medium-term survival outcomes in published studies; however, longer-term follow-up and additional high-quality clinical evidence are still required to confirm long-term predictability and stability | 5 (4-5); 95.2 | Yes (round 2) | Participants agreed that current short- to medium-term findings are favorable but emphasized the need for studies with longer follow-up, particularly beyond 5–10 years. Some noted that isolated long-term case reports and series already exist but provide low-level evidence. Implant design and manufacturing quality were considered potentially important sources of variability, while soft-tissue management was highlighted as a major determinant of long-term biological success | **Retained as final consensus statement** |
| **2. Early Postoperative Complications**  Early postoperative complications  (≤14 days), such as pain, swelling, and discomfort, are reported in  approximately 25–30% of cases and are generally mild, self-limiting, and  associated with minimal impact on implant survival | 4 (4-5); 87.0 | Not revised | Not presented in round 2 | - | Yes (round 1) | - | **Retained as final consensus statement** |
| **3. Late Biological Complications (1)**  Late biological complications (>14 days), including infection, soft tissue dehiscence, and chronic pain, occur in approximately 9–15% of patients, with implant removal required in  fewer than 5% of cases | 3.5 (2-4.25); 50.0 | Substantially revised to remove fixed complication rates and acknowledge that incidence and severity vary according to implant design, surgical technique, follow-up duration, and reporting methodology | **3. Late Biological Complications (1)** Late biological complications associated with patient-specific subperiosteal implants, including infection, soft tissue dehiscence, and chronic discomfort or pain, have been reported in the literature; however, the incidence and severity of these complications appear to vary according to implant design, surgical technique, follow-up duration, and reporting methodology, and further long-term clinical data are required | 5 (4-5); 100.0 | Yes (round 2) | Participants emphasized that late biological complications may be strongly influenced by patient selection, smoking, soft-tissue biotype, and peri-implant soft-tissue management. They also noted substantial variability among implant designs and manufacturers, cautioning against treating all patient-specific subperiosteal implants as a uniform device category | **Retained as final consensus statement** |
| **4. Late Biological Complications (2) / Locking Screw Systems**  To prevent screw loosening during  PSI fixation, a locking screw system can be considered. | 2.5 (1-3); 52.2 | Substantially revised to restrict locking screws to selected implant designs where implant thickness and anatomical location permit their use and where additional fixation stability is required | **4. Late Biological Complications (2)** Locking screw systems may be considered in selected patient-specific subperiosteal implant designs where implant thickness and anatomical location permit their use, particularly when additional fixation stability is desired. | 3 (3-4); 47.4 | No (round 2) | Participants remained divided regarding locking screw systems. Several emphasized that passive and intimate implant adaptation to the underlying bone is the primary objective and expressed concern that locking screws could lift the framework from the bone or introduce internal stresses. Others supported individualized selection of conventional or locking screws according to implant design, bone quality, anatomical location, and required fixation stability | **Discuss**  **No consensus achieved after Round 2.**  The disagreement appears substantive rather than purely semantic. The panel disagrees about whether locking screws provide meaningful benefit or might compromise passive fit  Possible wording for consensus-meeting discussion  **The fixation strategy for patient-specific subperiosteal implants should be individualized according to implant design, anatomical location, bone quality, framework thickness, and the ability to maintain passive implant adaptation. Locking screws may be considered when they provide a defined fixation advantage without compromising passive fit** |
| **5. Partial Implant Exposure**  Partial implant exposure is observed in  approximately 27–37% of subperiosteal implants but does not necessarily compromise long-term implant survival or prosthetic function  when appropriately managed | 4 (3-5); 73.9 | Revised to remove the fixed exposure rate, avoid an unsupported claim about long-term survival, and add implant-, anatomical-, material-, and patient-related qualifications. | **5. Partial Implant Exposure** Partial implant exposure has been reported in subperiosteal implants and may not necessarily compromise medium-term implant survival or prosthetic function when appropriately managed; however, outcomes may vary according to implant design, material, anatomical location, and patient-related factors. | 5 (4-5); 81.0 | Yes (round 2) | Participants supported the distinction between partial exposure and implant failure. Minor exposure was considered potentially manageable without compromising implant stability or prosthetic function, particularly when promptly addressed. Soft-tissue management and implant design were emphasized as important factors in preventing and managing this complication. | **Retained as final consensus statement** |
| **6. Mechanical Complications**  Mechanical complications, including  prosthetic disconnection, implant mobility, or minor prosthetic fractures, are generally uncommon and typically minor, with reported rates of up to 20%, and rarely necessitate implant removal | 4 (3-4); 73.9 | Substantially revised to remove the fixed complication rate and characterize reported mechanical complications as generally infrequent and manageable, while acknowledging limited evidence. | **6. Mechanical Complications** Mechanical complications, including prosthetic disconnection, implant mobility, or minor prosthetic fractures, have been reported in patient-specific subperiosteal implant systems. Available evidence suggests these complications are generally infrequent and manageable, although the current evidence remains limited and additional long-term studies are required | 4 (3-5); 52.4 | No (round 2) | Participants questioned whether the frequency of mechanical complications had been overstated and emphasized that different events should not be treated as equivalent. Prosthetic disconnection or minor prosthetic fracture may be manageable maintenance events, whereas implant mobility represents a major complication or treatment failure that may require implant removal. Modular and replaceable prosthetic components were viewed as potentially facilitating management of technical complications without removal of the implant framework. | **Discuss and substantially refine**  **No consensus achieved after Round 2**  The central problem is that the statement combines **minor prosthetic complications** with **implant mobility**, which participants regarded as a major failure.  Recommended approach  Split the statement into two concepts:  **6a. Prosthetic or technical complications**  **Technical complications, including prosthetic disconnection, screw-related complications, or minor prosthetic fractures, have been reported in patient-specific subperiosteal implant rehabilitation and may often be managed through prosthetic maintenance, repair, or replacement of modular components. However, the available long-term evidence remains limited**  **6b. Implant mobility**  **Mobility of a patient-specific subperiosteal implant framework represents a major mechanical complication requiring prompt clinical assessment and may necessitate implant revision or removal** |
| **7. Prosthesis Type Comparison**  Survival and complication rates are comparable between full-arch and bridge prostheses in both the maxilla  and mandible, with most studies reporting survival rates of 94% or higher regardless of prosthesis type | 4 (2.75-4); 65.2 | Substantially revised to remove the claim of comparable outcomes and instead acknowledge high reported survival for both prosthesis types while emphasizing the lack of robust direct comparative evidence. | **7. Prosthesis Type Comparison** Both full-arch and bridge prostheses supported by patient-specific subperiosteal implants have demonstrated high survival rates in published studies, generally exceeding 90%, although direct comparisons between prosthesis types remain limited due to heterogeneity in study designs, follow-up periods, and outcome reporting. | 4 (4-5); 90.5 | Yes (round 2) | Participants supported the revised statement but emphasized that the evidence base for segmental or bridge-supported rehabilitation is considerably smaller than that for full-arch treatment. Prosthesis selection should be guided by patient-specific anatomical and functional requirements, and the available evidence does not currently support firm direct comparisons between prosthesis types. | **Retained as final consensus statement** |
| **8. Outcomes in Complex Cases**  Subperiosteal implants used in  revision cases, post-oncological reconstructions, medication-related  osteonecrosis of the jaw, or congenital craniofacial anomalies  demonstrate similarly high survival rates with low complication rates, supporting their use in complex anatomical and medically compromised  cases | 4 (1.75-5); 56.5 | Substantially revised to report favorable outcomes in small case series without claiming consistently high survival or low complication rates and to explicitly acknowledge the limited and heterogeneous evidence base. | **8. Outcomes in Complex Cases** Subperiosteal implants have been used in complex clinical scenarios, including revision cases, post-oncological reconstructions, medication-related osteonecrosis of the jaw, and congenital craniofacial anomalies, with reported favorable outcomes in small case series. However, the current evidence base remains limited and heterogeneous, and further larger-scale and long-term studies are required to confirm their predictability in these indications | 5 (4-5); 90.5 | Yes (round 2) | Participants supported the potential value of patient-specific subperiosteal implants in complex anatomical situations where conventional augmentation or endosseous implant treatment may involve greater morbidity or limited predictability. The comments were generally supportive but did not provide additional evidence beyond individual clinical experience and published case-based observations. | **Retained as final consensus statement** |
| **9. Patient-Reported Outcomes**  Patient-reported outcomes, including comfort, function, aesthetics, and oral health-related quality of life, are  consistently positive following digitally planned subperiosteal implants, with reported improvements in chewing, speech, lip support, and facial aesthetics | 4 (3.75-5); 78.3 | Not revised | Not presented in round 2 | - | Yes (round 1) | - | **Retained as final consensus statement** |

Theme 5: Follow-Up and Maintenance

| **Original statement** | **Round 1 results**  **Median (IQR); Agreement (%)** | **Modification after round 1** | **Statement presented in round 2** | **Round 2 results**  **Median (IQR); Agreement (%)** | **Consensus** | **Round 2 comments** | **Final decision (retained, minor editorial refinement, discuss, exclude)** |
| --- | --- | --- | --- | --- | --- | --- | --- |
| **1. Structured Follow-Up Protocols**  Patient-specific implant-supported  prostheses should be monitored using structured follow-up protocols,  including regular clinical and radiographic assessments, particularly  during the first 12 months after placement to detect early biological or  mechanical complications | 5 (4-5); 87.0 | Not revised | Not presented in round 2 | - | Yes (round 1) | - | **Retained as final consensus statement** |
| **2. Early Postoperative Monitoring**  Close postoperative follow-up during  the first weeks after surgery is essential to monitor soft tissue healing,  implant stability, and patient comfort, as most early complications are identified during this period | 4 (3.75-5); 78.3 | Wording refined to emphasize that the first postoperative weeks are critical for the active detection and management of early complications | **2. Early Postoperative Monitoring** Close postoperative follow-up during the first weeks after surgery is recommended to monitor soft tissue healing, implant stability, and patient comfort, as this period is critical for detecting and managing early postoperative complications | 5 (5-5) 90.5 | Yes (round 2) | Participants strongly supported structured early postoperative monitoring. One participant suggested specifying a clinically relevant early follow-up period of approximately 10–14 days because soft-tissue complications may emerge after the initial postoperative review. Another described a structured protocol incorporating early soft-tissue assessment, professional cleaning, provisional-prosthesis review, and subsequent planning for the definitive restoration | **Retained as final consensus statement** |
| **3. Soft Tissue and Peri-implant Assessment**  Routine follow-up visits  should include assessment of peri-implant soft tissues, including signs of  inflammation, bleeding, mucosal recession, or implant exposure, as  these parameters are critical indicators of long-term implant success | 4.5 (4-5); 87.0 | Not revised | Not presented in round 2 | - | Yes (round 1) | - | **Retained as final consensus statement** |
| **4. Radiographic Surveillance**  Periodic radiographic evaluation is  recommended to assess implant positioning, fixation stability, and the absence of pathological bone changes, particularly in full-arch and complex reconstructions | 4 (3.75-5); 78.3 | Revised from periodic routine surveillance to selective radiographic evaluation guided by clinical findings, while acknowledging the limited diagnostic value of imaging in some patient-specific subperiosteal implant cases | **4. Radiographic Surveillance** Radiographic evaluation should be performed selectively and guided by clinical findings to assess implant positioning, fixation stability, and potential pathological bone changes, particularly in full-arch and complex reconstructions, as routine imaging may have limited diagnostic value in the presence of patient-specific subperiosteal implants | 5 (4-5); 90.5 | Yes (round 2) | Participants agreed that clinical examination, soft-tissue assessment, prosthetic stability, and patient-reported symptoms are central to follow-up, but opinions differed regarding the frequency of radiographic imaging. Some supported selective imaging based on clinical indications, whereas others advocated routine CBCT or panoramic imaging at defined postoperative and long-term intervals. Proposed protocols ranged from immediate postoperative imaging and short-term assessment to annual CBCT during the first 4–5 years or less frequent imaging thereafter | **Minor editorial refinement.**  Reason  The revised statement achieved strong consensus, but the comments show continuing disagreement about whether imaging should be exclusively symptom-driven or whether scheduled baseline and periodic radiographs have value. The current phrase **“performed selectively and guided by clinical findings”** may be interpreted as excluding planned baseline surveillance.  Suggested refined wording  **Radiographic evaluation should be individualized according to the clinical situation, implant design, reconstruction complexity, and patient-specific risk factors. Baseline postoperative imaging and subsequent radiographic assessment may be considered to evaluate implant position, fixation, and potential pathological bone changes, while additional imaging should be guided by clinical findings and recognition that patient-specific subperiosteal frameworks may limit the diagnostic value of some radiographic examinations** |
| **5. Maintenance and Hygiene Protocols**  Long-term maintenance of  patient-specific implants requires individualized oral hygiene instructions  and scheduled professional maintenance visits to support peri-implant health and minimize biological complications | 5 (5-5); 100.0 | Not revised | Not presented in round 2 | - | Yes (round 1) | - | **Retained as final consensus statement** |
| **6. Management of Biological Complications**  Biological complications  such as soft tissue infections or implant exposure should be addressed promptly through hygiene protocols, antibiotics, or prosthetic  adjustments to maintain functional outcomes and prevent implant loss | 5 (4-5); 95.7 | Not revised | Not presented in round 2 | - | Yes (round 1) | - | **Retained as final consensus statement** |
| **7. Prosthetic Maintenance and Adjustments**  Regular prosthetic  evaluation during follow-up is necessary to identify occlusal wear, screw  loosening, or minor prosthetic complications, which can usually be  managed with simple maintenance or prosthetic adjustments | 5 (4-5); 91.3 | Not revised | Not presented in round 2 | - | Yes (round 1) | - | **Retained as final consensus statement** |
| **8. Importance of Long-Term Follow-Up**  Long-term follow-up is essential to evaluate implant survival, functional stability, and patient-reported outcomes, as mid- to long-term evidence for digitally manufactured  patient-specific implants continues to evolve | 5 (4-5); 100.0 | Not revised | Not presented in round 2 | - | Yes (round 1) | - | **Retained as final consensus statement** |
